# Implementation of a Ponseti Clubfoot Program Decreases Major Surgery: A Quality Improvement Initiative

**DOI:** 10.1097/pq9.0000000000000362

**Published:** 2020-10-23

**Authors:** Patrick M. Carry, Susan Graham, Karen Whalen, Deborah Burke, Robin Baschal, Kaley S. Holmes, Brian Kohuth, Gaia Georgopoulos, Nancy Hadley Miller

**Affiliations:** From the * Musculoskeletal Research Center, Children’s Hospital Colorado, Aurora, Colo.; † Department of Orthopedics, University of Colorado Denver Anschutz Medical Campus, Aurora, Colo.

## Abstract

Clubfoot describes a congenital condition. If untreated, clubfoot can cause long-term functional issues. The Ponseti method is the gold-standard treatment; it emphasizes casting over surgery. We identified a high rate of major recurrence in patients with isolated clubfoot at our institution. We implemented a quality improvement intervention to address the recurrences.

**Methods:**

We established a clubfoot program that aimed to (1) develop a standardized treatment pathway; (2) improve care team education; (3) improve coordination of care with families; and (4) improve documentation. The purpose of this study was to outline our quality improvement intervention and evaluate its success. Data were retrospectively collected from isolated clubfeet before (2003–2007, phase I) and after (2012–2014, phase II) implementation of the clubfoot program. We compared the differences in treatment and major recurrence between the 2 phases using generalized logistic or linear mixed models. Modified Poisson regression models were used to evaluate the association between provider nonadherence and recurrence.

**Results:**

The pre- (phase I) and post- (phase II) implementation groups included 91 patients (131 feet) and 68 patients (101 feet), respectively. The incidence of major recurrence (odds ratio: 59.5, 95% confidence interval: 7.8–454.4, *P* < 0.0001) was lower during phase II compared to phase I. Nonadherence with the care pathway was associated with an increased risk of recurrence (risk ratio: 4.1, 95% confidence interval: 1.2–14.3, *P* = 0.0274).

**Conclusions:**

The implementation of a clubfoot program was associated with a decrease in major clubfoot surgery and improved adherence to established guidelines for clubfoot management.

## Abstract 2

Supplemental Digital Content is available in the text.

## INTRODUCTION

Quality improvement (QI) initiatives address the medical community’s responsibility to remain accountable for interventions and outcomes. Assessing the outcomes of QI interventions is problematic due to the variable presentation of medical disease and the practitioner’s responsiveness based on training, expertise, and the practice environment. Although these factors are significant, QI initiatives are essential in the effort to improve healthcare value.^1,2^

QI and process improvement initiatives are becoming common in orthopedics, lead by the American Academy of Orthopaedic Surgeons (AAOS).^2–7^ The AAOS Evidence-Based Medicine Unit has developed over 20 clinical practice guidelines covering topics from arthroplasty to supracondylar elbow fractures.^8^ Orthopedic-related QI initiatives have achieved positive outcomes including a decrease in trauma surgery wait time,^3^ prevention of secondary osteoporotic fractures,^9^ a decrease in pediatric cast saw injuries,^10^ a decrease in length of stay and operative time for hip fractures,^11^ and lower cost for total knee replacement surgery.^12,13^

The QI process addresses a clinical issue, diagnosis, or theme, where it may increase the value in clinical- and financial-based outcomes.^2,7^ Interventions respond to gaps in the quality and/or cost of care relative to benchmarks or expectations. At our institution, we observed an unacceptable, high rate of major surgical events for the correction of isolated clubfoot.^14^ Subsequently, we initiated a QI intervention to address gaps in healthcare delivery within our institution relative to our goals. We identified the system gaps and developed our internal goals by reviewing treatment metrics at our institution relative to a renowned clubfoot treatment program.^14^ We aimed to address gaps that were different between institutions and/or inconsistent with Ponseti’s best practices.^15^ The QI intervention established a Ponseti-based clubfoot program that incorporated a clinical care pathway. The program also included trained providers, a specialized support staff, trained physical therapists and casting technicians, and monetary support for family education materials, website development, and individualized family support. The purpose of this study was to evaluate our QI intervention and determine if provider pathway adherence was associated with major recurrence.

## METHODS

### Program Development and Implementation

**Fig. 1.**
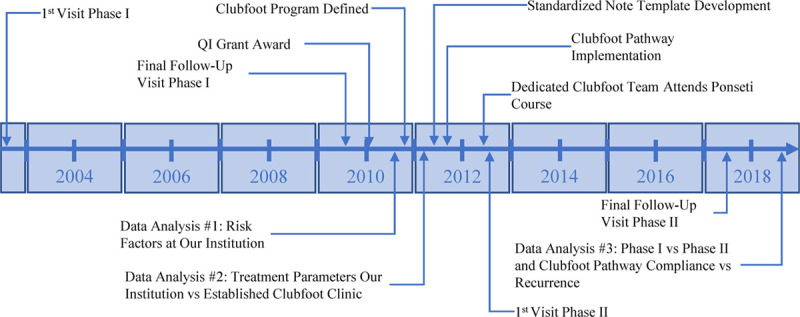
QI intervention timeline. The figure summarizes all relevant events during problem identification (phase I analysis #1 and #2), intervention development, and intervention evaluation (data analysis #3). The x axis summarizes time beginning with the first cast visit during phase I to the data analysis and dissemination components of phase II.

Our Ponseti clubfoot program included the following: (1) identification of system gaps by reviewing treatment metrics at our institution relative to a well-established program^14^; (2) piloting change through the development of a clinical care pathway^2^; (3) implementing change through dedicated staff, institutional support, and specialized training; (4) institution of data processes to track progress and outcomes; and (5) systematic responsiveness to hurdles impeding success. The culmination of the process was developing a clinical care pathway and a dedicated clubfoot team (Fig. 1).

The first and most crucial step in the process was to present the poor outcomes (high rate of major surgery) to our department. The department and hospital then agreed to fund a clubfoot program. Everyone in our department was invited to join the clubfoot team if they agreed to our stipulations (1) become trained in the Ponseti technique (see https://globalclubfoot.com/ and http://www.ponseti.info/mentorship-program) and (2) adhere to the clubfoot treatment pathway. Our team included orthopedic surgeons, advanced practice providers (APPs), physical therapists, orthopedic cast technicians, orthotists, and registered nurses. Once we established our team, we developed the clinical care pathway. Table 1 outlines the components comprising the pathway, rationale for each component, and metric(s) used to assess adherence during phase II. We also established consistent guidelines for the casting visits; all foot manipulations were done and held by the orthopedic surgeon while a technician applied the cast. The orthopedic surgeon then did the molding. Educational materials were developed for families to aid in bracing compliance. Consistent with the objectives of a clinical care pathway,^2^ the goal of our pathway was to reduce treatment variation and improve care coordination. A nurse coordinator monitored the program. The coordinator was essential in identifying provider and patient-related barriers and pathway deviations, communicating these issues to the team, and resolving them. For example, one family’s schedule prevented them from attending a routine clubfoot clinic with full evaluation from the team’s physical therapist. Therefore, arrangements were made for an isolated therapist visit outside of the regular clinic arrangement.

**Table 1.** Pathway Components, Rationale, and Metric(s) Used to Assess Each Component

Component	Rationale	Metric*
(1) Serial casts applied weekly	Number of casts and time between casts was increased in phase 1.	Average time between casts
	Decreasing time between casts decreases overall treatment course and ensures treatment is consistent and adheres to Ponseti best practices	& number of casts
(2) Serial casts applied by no more than 2 providers	Number of providers was very high during phase 1.	Proportion of feet treated by 3 or more providers
	By decreasing number of providers, we aimed to improve continuity of care and build patient/family trust.	
(3) Cast applied every single visit	Incidence of cast complications was very high during phase 1.	Incidence of cast complications
	Cast complications lead to break in casting that can prolong casting period and potentially allow minor recurrences to occur.	
(4) Patient is evaluated by Orthopedic surgeon within 4 weeks of cast initiation† and (5) Tenotomy within 8 wks of the first cast	Rate of tenotomies was very low during phase 1.We aimed toevaluate patient for tenotomy early in treatment so that procedure is scheduled in a timely manner.	Proportion of patients that underwent a tenotomy
		Time to first visit with a surgeon
		Time to tenotomy
(6) Ponseti brace full time for 3 mo, followed by part-time wear until 4 to 5 yrs of age	Brace compliance is essential to long-term success.	Proportion of feet compliant with bracing recommendations during first 2 years‡
	Provide consistent education for families regarding importance of bracing minimize deformity recurrence.	

*All measures were selected based on data available in the EMR that had been incorporated into the standardized note templates during phase 2 implementation.

†The orthopedic surgeon evaluated the patient at their initial visit, and then at least one other time within 4 wks of cast initiation.

‡We limited assessment of compliance to the first 2 yrs only. Long-term bracing compliance was beyond the scope of this initial study meant to decrease incidence of major surgery within 2 yrs of casting.

### Program Evaluation

We collected data retrospectively from a consecutive series of patients with isolated clubfoot pre-implementation (January 2003 to December 2007, n = 131 feet, phase I) and post-implementation (March 2012 to March 2015, n = 101 feet, phase II, Fig. 1) of the clubfoot program. Variables related to pathway components (Table 1), demographics, and recurrences were collected. We defined a minor recurrence as any recurrence that resulted in deviations from the routine follow-up, including repeat casting, a revision tenotomy, and/or an anterior tibialis tendon transfer. We defined a major recurrence as any recurrence that required extensive soft tissue release surgery or bony procedures beyond repeat tenotomy and/or tendon transfers. We classified cast complications as any complication that caused a break in casting, such as skin sores or cast slipping. We specified brace noncompliance as provider documentation of noncompliance with the bracing prescription on one or more clinic visits. During phase I, 2 researchers collected the data. The team developed a set of definitions for all pathway components and met weekly to review data accuracy. The PI met with the team as necessary to resolve any issues.

In phase II, a single researcher collected data. The study team reviewed any data collection issues at a monthly meeting. Among subjects in phase II, we also assessed provider adherence. We evaluated adherence for 5 of the components (see Table 1). We defined pathway adherence (adherent versus non-adherent for the 5 components at the patient level, allowing us to test the association between nonadherence and recurrence (Table 1). We did not evaluate adherence to the sixth component due to a lack of reliable 4- to 5-year follow-up and inconsistent documentation of the timing of nonadherence relative to the timing of recurrence. Data were collected retrospectively during both study phases. The study was reviewed and approved by our quality review committee.

### Sample Size Calculation:

Based on the enrollment (n = 131 feet, 91 patients) and major recurrences (45%) in phase I,^14^ adequate sample size was determined to be 60 patients or 90 total feet in phase II. This number would provide 90% power, at an alpha level of 0.05, to reject the null hypothesis of no difference in the incidence of major recurrence between phases. This sample size calculation conservatively assumed a 25% incidence of major recurrence in the phase II group [odds ratio (OR) of 2.5], a within-patient correlation of 0.45, and an average of 1.5 clubfeet per subject (50% prevalence of bilateral clubfeet).

### Statistical Methods

**Fig. 2.**
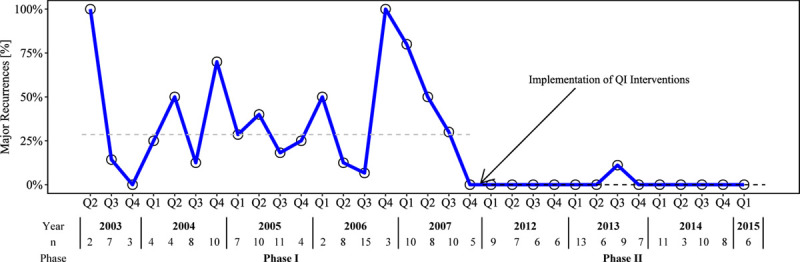
Run chart of major recurrence. The x axis represents time, quarter, and the y axis represents the outcome during phases I and II. The dashed lines represent the median incidence of major recurrence during phase I (29%, gray line) and phase II (0%, black line). A lower incidence of major recurrence represents a positive outcome. The sample size, number of feet, in each quarter is displayed in the table below the figure.

We used Chi-square and Student’s t-tests to compare the demographics and clinical characteristics in the 2 phases. Generalized logistic and linear mixed models were used to test for group differences in the binary and continuous treatment pattern outcomes, respectively. We used generalized logistic regression analyses to test for differences in the incidence of major recurrence, as well as the incidence of minor recurrence and/or major recurrence between the 2 groups. We used generalized estimated equations to account for clustering due to the inclusion of subjects with bilateral isolated clubfeet. To minimize bias due to differences in follow-up duration between the 2 cohorts, we only considered recurrences during the first 2 years after casting. We also created run charts to augment the primary analysis (**see figs. 2–8, Supplemental Digital Content 1,** http://links.lww.com/PQ9/A219. We used a modified Poisson regression analysis to estimate the association between provider adherence with the Ponseti-based clinical care pathway components and the risk of minor or major recurrence. We assessed adherence at the patient level (one measurement per subject) due to the uniform consistency in adherence among subjects affected by bilateral clubfoot. Among the bilateral subjects, we included the worse outcome of the two feet in the association between adherence and recurrence. We adjusted for age in all analyses.

## RESULTS

A total of 91 patients (131 feet) and 68 patients (101 feet) were included in phases I and II, respectively. We excluded subjects treated at an outside hospital, affected by an underlying neuromuscular or syndromic condition, not affected by clubfoot, followed for less than two years, or missing treatment information (s**ee fig. 1, Supplemental Digital Content 1,** **http://links.lww.com/PQ9/A219**). The purpose of the enrollment criteria was to exclude atypical clubfoot and older subjects, factors associated with a more challenging treatment regimen, and factors that would prevent us from accurately assessing pathway components (see Table 1) and/or outcomes. The 2 cohorts were similar for gender, primary language, positive family history, perinatal complications, age at first casting, and average birth weight (Table 2).

**Table 2.** Limb Level Demographics and Clinical Characteristics

	Phase I		Phase II		*P*
	131 Feet		101 Feet		
Female gender, N (%)	30	23%	30	30%	0.3516
English as primary parental language	112	85%	89	88%	0.4436
Positive family history, N (%)	32	24%	26	26%	0.9050
Perinatal complications, N (%)	15	11%	19	19%	0.2423
Bilateral, N (%)	81	62%	66	65%	0.6641
Average birth weight (lbs), mean (stdev)	6.7	1.6	7.1	1.1	0.1290
Age baseline (wks), mean (stdev)	1.6	1.2	1.5	0.9	0.2179

In phase II, there was a change in pathway components (Table 3). Cast complications [OR: 3.4, 95% confidence interval (CI): 1.6–7.5, *P* = 0.0023] were more frequent during phase I. Subjects were also more likely to be treated by three or more providers during phase I (OR: 6.7, 95% CI: 2.2–20.3, *P* = 0.0008). There was an increase in the average days between casts (mean difference: 2.5 days, 95% CI: 2.0–3.0, *P* < 0.0001) and the average number of casts (mean difference: 2.8, 95% CI: 1.8–3.8, *P* < 0.0001) during phase I. During phase I, subjects were less likely to receive a tenotomy (OR: 0.3, 95% CI: 0.2–0.7, *P* = 0.0055).

**Table 3.** Treatment Components by Phase

	Phase I		Phase II		*P*
	n = 131 feet		n = 101 feet		
Noncompliance during bracing, N (%)	48	37%	51	50%	0.0275*
Underwent tenotomy, N (%)	86	66%	86	85%	0.0055*
Complication noted during a cast visit, N (%)	49	37%	16	16%	0.0023*
Revision cast, N (%)	20	15%	11	11%	0.2945*
Multiple providers during casting, N (%)	36	27%	6	6%	0.0008*
Average number of casts, mean (stdev)	8	3.4	5.7	2.5	<0.0001†
Weeks between casts, mean (stdev)	8	1.8	5.6	1	<0.0001†

*P value based on the generalized logistic regression analysis, adjusted for age at first cast.

†*P* value based on linear mixed model, adjusted for age at first cast.

The run charts supported the primary analysis. There was an improvement in the incidence of major (Fig. 2) and minor recurrence **(see fig. 2, Supplemental Digital Content 1,** http://links.lww.com/PQ9/A219), the proportion of feet that underwent a tenotomy (**see fig. 7, Supplemental Digital Content 1,** http://links.lww.com/PQ9/A219), and average days between casts (**see fig. 5, Supplemental Digital Content 1,** http://links.lww.com/PQ9/A219) during phase II. There was temporal variability in some of the outcome variables, including the proportion of feet treated by three or more providers during Phase II (**see fig. 4, Supplemental Digital Content 1,** http://links.lww.com/PQ9/A219). This trend is due to an increased emphasis on provider adherence championed by the nurse coordinator. There was also variability in the number of casts across both study phases (**see fig. 6, Supplemental Digital Content 1,** http://links.lww.com/PQ9/A219). Unexpectedly, bracing compliance was higher during phase I compared to phase II (OR: 0.6, 95% CI: 0.3–0.9, *P* = 0.0275, **see fig. 8, Supplemental Digital Content 1,** http://links.lww.com/PQ9/A219). We suspect it is due to noncompliance in combination with better documentation. During phase II, documentation of noncompliance with bracing was built directly into the standardized notes. In contrast, compliance with bracing was documented with less consistency during phase I.

In phase II, there was a decrease in the incidence of major (33.6%, 44/131 versus 1.0%, 1/101) and minor recurrence (40.5%, 53/131 versus 11.9%, 12/101). After adjusting for age, the odds of major recurrence during phase I was 59.5 (95% CI: 7.8–454.4, *P* < 0.0001) times the odds of major recurrence in phase II. The odds of a major or minor recurrence were also higher in phase I (OR: 5.0, 95% CI: 2.2–11.3, *P* < 0.0001). The run charts support this conclusion, demonstrating a temporal, downward shift in the incidence of major recurrence (Fig. 2) as well as major and minor recurrence (**see appendix 2, Supplemental Digital Content 1,** http://links.lww.com/PQ9/A219) during phase II. In Figure 2, the incidence of major recurrence for each quarter in phase II was less than median incidence of recurrence during phase I, indicating a consistent decrease in major recurrences after the QI intervention.

Providers were most compliant with component 4 and least compliant with component 5 (Table 4). The risk of a minor or major recurrence when providers were noncompliant with one or more components was 4.1 (95% CI: 1.2–14.3, *P* = 0.0274) times the risk of a minor or major relapse when providers were compliant will all 5 components.

**Table 4.** Association between Provider Compliance and Risk of a Minor or Major Recurrence

Component		Compliance	Risk Ratio*	95% CI	*P*
(1)	Serial casts applied weekly: noncompliant vs. compliant	91.2% (62/68)	2.8	0.7–11.3	0.1371
(2)	Serial casts applied by no more than 2 providers: noncompliant vs. compliant	94.1% (64/68)	4.6	1.4–14.4	0.0099
(3)	Cast applied at every visit (avoidance of breaks due to cast complications): noncompliant vs. compliant	95.6% (65/68)	2.5	0.4–15.4	0.3196
(4)	Patient is evaluated by an orthopedic surgeon within 4 wks of cast initiation: noncompliant vs. compliant	97.1% (66/68)	9.4	4.6–19.4	<0.0001
(5)	Tenotomy within 8 wks after cast initiation: noncompliant vs. compliant	88.2% (60/68)	6.0	1.8–20.0	0.0035

*Risk ratios estimated at the patient level due to lack of within subject variability in provider compliance at the limb level.

## DISCUSSION

In response to an initial review of clubfoot care within our institution, we implemented a QI initiative to establish a dedicated, the Ponseti-based clubfoot program to decrease major recurrence. The QI implementation process translated into tangible clinical improvements as demonstrated by an improvement in pathway components and a decrease in the major recurrences.

The development of the care pathway was an essential component of the clubfoot program. Implementation of this initiative required substantial effort from multiple domains and, in turn, created a 5-year gap between phases I and II. This time was necessary to gather retrospective data, acquire hospital and nursing support, provide education, and build partnerships. Additionally, isolated clubfoot is a heterogeneous condition. In contrast to clinical practice guidelines that adopt a more rigid treatment algorithm, clinical care pathways provide a consensus-based, standardized care model, but allow for variation in treatment methodology to meet the unique demands of the individual patient.^2^ The goal of a clinical care pathway is to standardize treatment through the implementation of best practice recommendations based on evidence in the current literature or care pathway team consensus.^2^ The process required input from relevant stakeholders including surgeons, physical therapists, nurses, APPs, information technology support concerning EMR template design and implementation, and administrative support staff. This process highlighted discrepancies in treatment approach and delivery. The development of consensus ensured that all providers shared a common vision.

The implementation of a dedicated Ponseti-based clubfoot program at our institution decreased major clubfoot surgery. The incidence of major recurrence during phase II (1%) was consistent with institutions with internationally recognized clubfoot programs.^14–18^ Of note, major surgery is based on physician discretion. The dramatic reduction could be attributed to changes in surgical indications, suggesting treatment was not necessarily getting better. Still, surgeons were more likely to explore less invasive treatment during phase II. If the change in the incidence of major surgery was due to misclassification of major recurrences as minor recurrences (treated with repeat casting), we would anticipate an increase in minor recurrences in phase II. However, we observed a decrease in all recurrences, minor (41% phase I versus 12% phase II), and major (34% phase I versus 1% phase II), following the clubfoot program’s implementation.

The Ponseti-based clinical care pathway components were developed based on the current literature, Ponseti tutorials, and treatment approaches at an institution with a well-established clubfoot program. There was a 76% reduction in the odds of recurrence when providers were compliant with the care pathway. This result indicates that implementing the clubfoot program not only changed clubfoot pathway components, but that adherence with the clinical care pathway resulted in superior outcomes.^13^

Postimplementation, a commitment to continued surveillance and the flexibility to intervene on new hurdles are critical components of the plan-do-check-act continuous improvement cycle. Adherence with the Ponseti-based clinical care pathway was high in all areas except bracing. Bracing noncompliance in phase II was close to 50% (47%), representing an opportunity for improvement. Previous research has identified bracing noncompliance as a significant predictor of treatment failure.^19^ It is natural to put the majority of the blame on parent/caregiver for lack of compliance and to conclude that noncompliance is a direct cause of recurrence. However, bracing noncompliance is a complex issue. It can be related to multiple factors, such as the severity of the deformity, gaps in provider/family communication, and access to and cost of bracing modifications. It is essential to consider the complexity of other factors, such as deformity severity that can make bracing intolerable. Gaps in communication between family and provider can also lead to noncompliance. It is crucial to consider the complexity of noncompliance and intervene in all modifiable aspects. We are exploring additional interventions to improve brace compliance by engaging the families and ensuring all relevant stakeholders understand the barriers to brace compliance. Consistent with the application of the patient- and family-centered collaborative care model to orthopedics,^1,7^ the goal of our next intervention is to create a refined vision of the ideal care experience, one that incorporates input from both providers and patients.

There are several limitations to this work. We implemented the intervention in a large pediatric hospital with ongoing support from the hospital and department leadership. The results and sustainability of this model may not be generalizable to other hospitals, especially those with less support for QI interventions. We did not record clubfoot outcomes during the period between the end of phase I and the start of phase II. Thus, we cannot exclude the possibility that temporal factors, external to the intervention, contributed to improvements in outcome during phase II.

Similarly, we did not screen for atypical clubfeet,^20^ which may have differed across the study periods. The incidence of major recurrence was based on a subjective determination of the need for major surgery. Bracing compliance is an essential aspect of clubfoot management. We tested for differences in bracing compliance between phases. We did not incorporate bracing compliance into our analysis of pathway compliance and recurrence due to incomplete documentation of timing of bracing noncompliance relative to the timing of recurrence. We did identify an association between pathway treatment non-compliance and risk of recurrence. However, based on the data collection mechanisms, we were not able to determine the root cause of each recurrence and/or identify aspects of the pathway that were driving the recurrences. Last, we did not measure cost. However, we are confident that the decrease in the average number of casts and the decrease in major recurrence translated to improvements in patient-related and insurance costs. Hospital-associated costs are more difficult to quantify as costs may be offset by intangibles, such as an improved reputation within the referral base of community providers.

## CONCLUDING SUMMARY

Ponseti casting is the international gold standard.^21^ However, clubfoot treatment is a challenging process that requires commitment from providers, healthcare facilities, and families. To address these challenges, our institution implemented an iterative QI process to establish a dedicated Ponseti clubfoot program. We designed a clinical care pathway with specific components based on evidence from the literature and anecdotal best-practice recommendations. Our intervention resulted in a decrease in the number of casts needed per foot, an increase in the number of feet treated by a single provider (improving continuity of care), an increase in the proportion of feet that underwent tenotomy, and a decrease in the incidence of major recurrence. Globally, we have provided a blueprint for our orthopedic department as well as others to develop sustainable high-value quality clinical programs by, first, identifying gaps in quality of care relative to national standards; second, critically reviewing the delivery of care and identifying modifiable factors that may contribute to this quality gap; third, developing an interventional program to address the gaps; and, last, implementing an ongoing assessment of the quality of care delivered by the program.

## DISCLOSURE

The authors have no financial interest to declare in relation to the content of this article.

## ACKNOWLEDGMENTS

The institution of the authors has received support from NIH/NCATS Colorado CTSA Grant Number UL1 TR002535. Contents are the authors’ sole responsibility and do not necessarily represent official NIH views. The authors have received funding from the Research Institute of Children’s Hospital Colorado in the form of a Quality and Systems Improvement grant in 2010.

## Supplementary Material


