# Supplementary material for: Implementation of a Ponseti Clubfoot Program Decreases Major Surgery: A Quality Improvement Initiative
Source: Pediatr Qual Saf. 2020 Oct 23;5(6):e362. doi: 10.1097/pq9.0000000000000362 (PMC7870159; doi:10.1097/pq9.0000000000000362)
Supplement: Supplementary file 1 [file pqs-5-e362-s001.pdf]

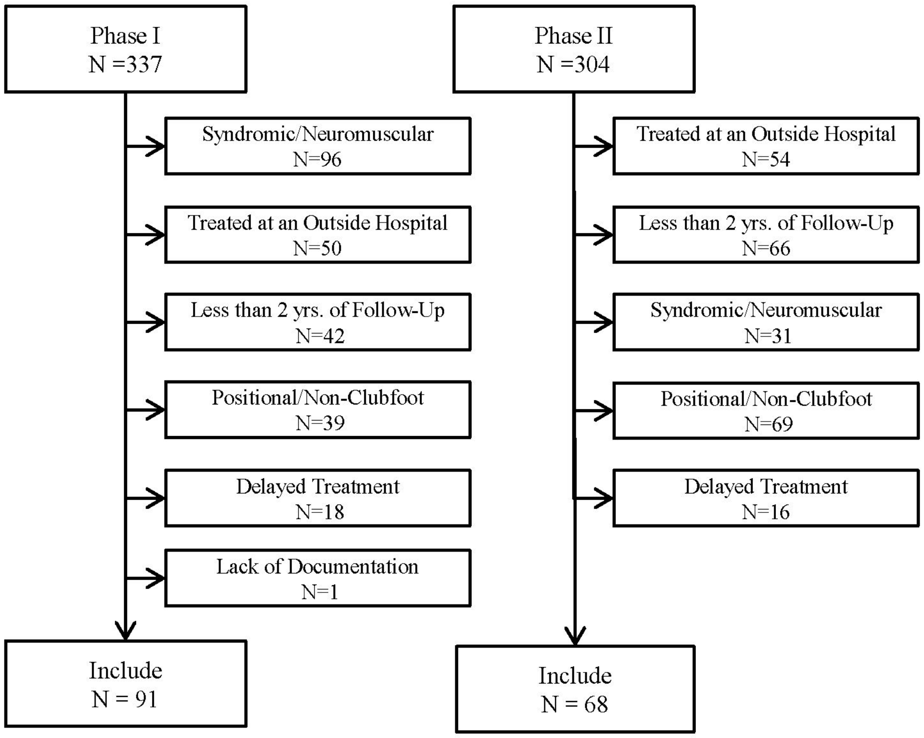
Phase I
N =337
Syndromic/Neuromuscular
N=96
Treated at an Outside Hospital
N=50
Less than 2 yrs. of Follow-Up
N=42
Positional/Non-Clubfoot
N=39
Delayed Treatment
N=18
Lack of Documentation
N=1
Include
N = 91
Phase II
N =304
Treated at an Outside Hospital
N=54
Less than 2 yrs. of Follow-Up
N=66
Syndromic/Neuromuscular
N=31
Positional/Non-Clubfoot
N=69
Delayed Treatment
N=16
Include
N = 68

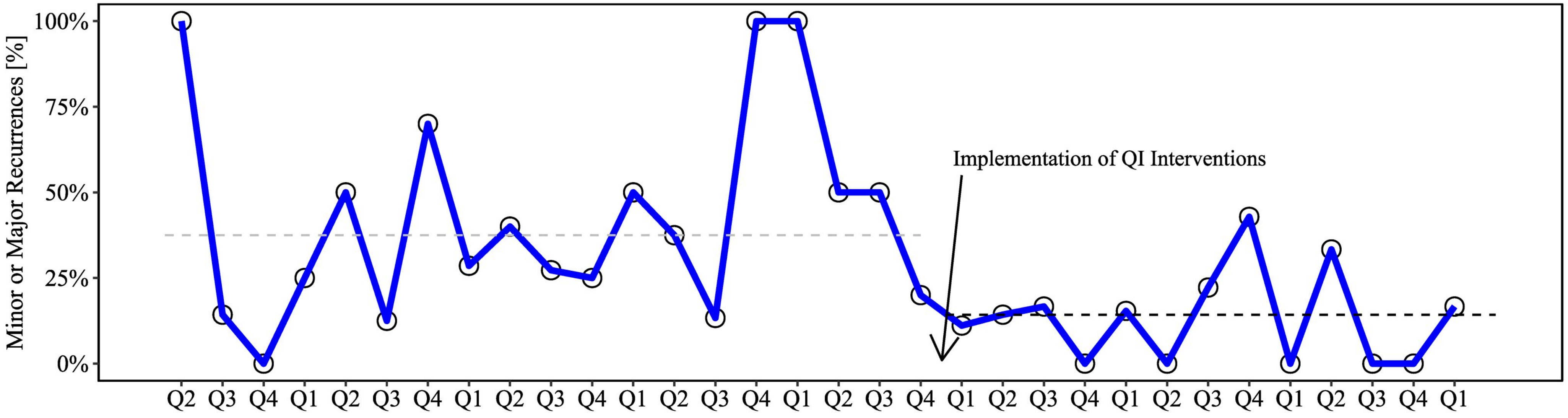

Year	**2003**			**2004**				**2005**				**2006**				**2007**				**2012**				**2013**				**2014**				**2015**
n	2	7	3	4	4	8	10	7	10	11	4	2	8	15	3	10	8	10	5	9	7	6	6	13	6	9	7	11	3	10	8	6
Phase	**Phase I**																			**Phase II**												

Figure Description: The X-axis represents time, quarter, and the Y-axis represents the outcome during Phase I and Phase II. The dashed lines represent the median incidence of minor or major recurrence during Phase I (38%, gray line) and Phase II (14%, black line). A lower incidence of minor or major recurrence represents a positive outcome. The sample size, number of feet, in each quarter is displayed in the table below the figure.

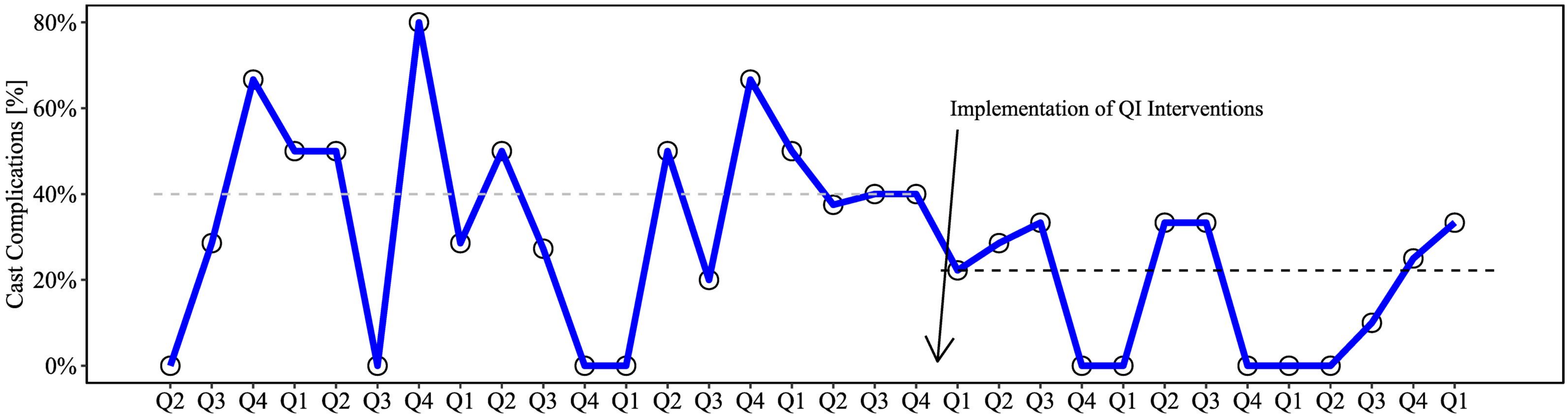

Year	2003	2004	2005	2006	2007	2012	2013	2014	2015
n	2 7 3	4 4 8 10	7 10 11 4	2 8 15 3	10 8 10 5	9 7 6 6	13 6 9 7	11 3 10 8	6
Phase			**Phase I**				**Phase II**		

Figure Description: The X-axis represents time, quarter, and the Y-axis represents the outcome during Phase I and Phase II. The dashed lines represent the median incidence of cast complications during Phase I (40%, gray line) and Phase II (22%, black line). A lower incidence of cast complications represents a positive outcome. The sample size, number of feet, in each quarter is displayed in the table below the figure.

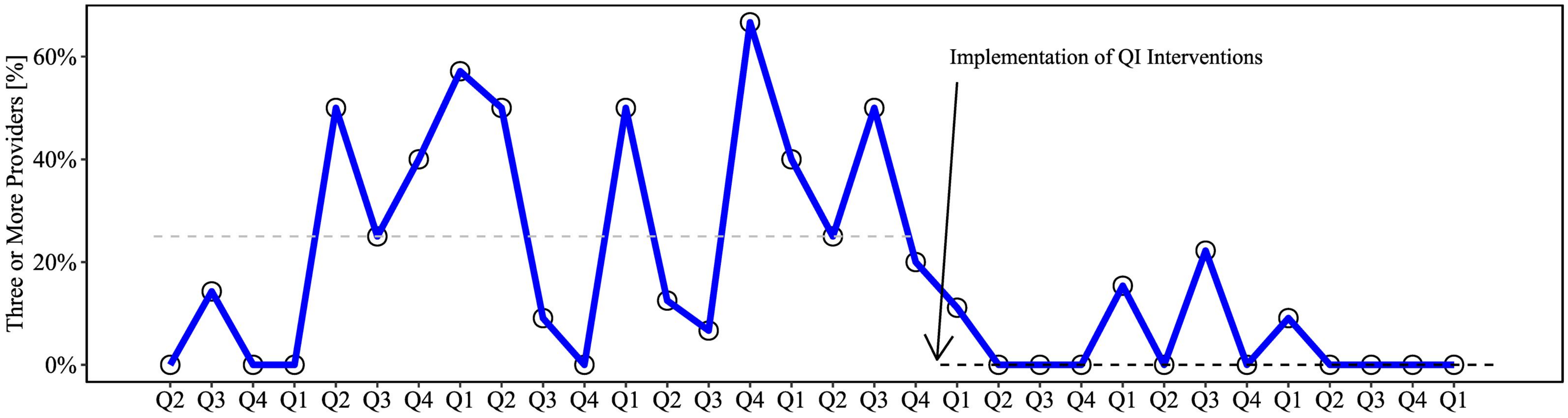

Year	**2003**			**2004**				**2005**				**2006**				**2007**				**2012**				**2013**				**2014**				**2015**
n	2	7	3	4	4	8	10	7	10	11	4	2	8	15	3	10	8	10	5	9	7	6	6	13	6	9	7	11	3	10	8	6
Phase	**Phase I**																			**Phase II**												

Figure Description: The X-axis represents time, quarter, and the Y-axis represents the outcome during Phase I and Phase II. The dashed lines represent the median percentage of feet treated by three or more providers during Phase I (25%, gray line) and Phase II (0%, black line). A lower percentage of feet treated by three or more providers represents a positive outcome. The sample size, number of feet, in each quarter is displayed in the table below the figure.

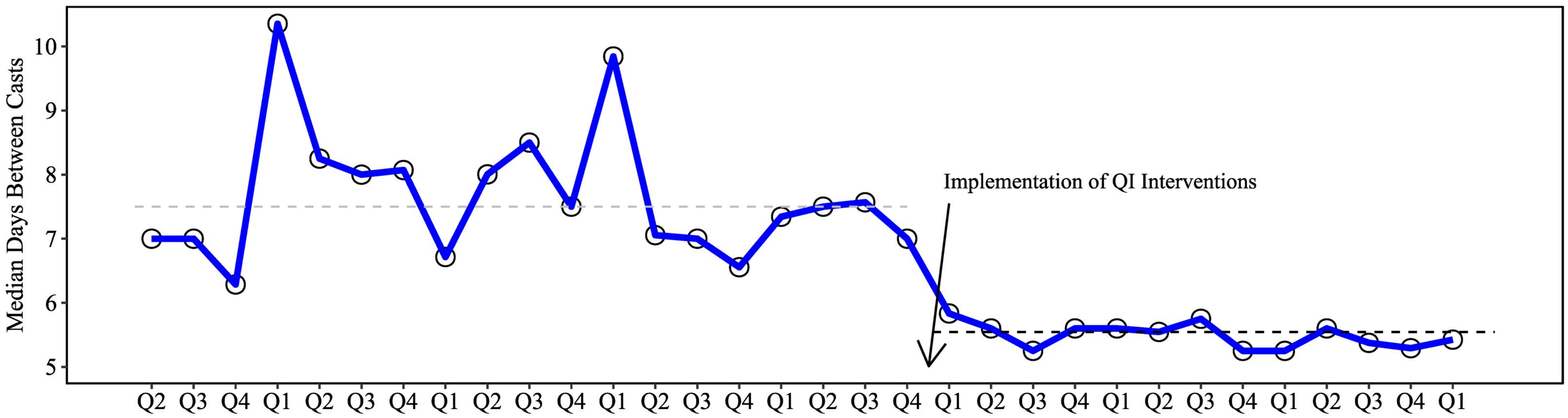

Year	**2003**			**2004**				**2005**				**2006**				**2007**				**2012**				**2013**				**2014**				**2015**
n	2	7	3	4	4	8	10	7	10	11	4	2	8	15	3	10	8	10	5	9	7	6	6	13	6	9	7	11	3	10	8	6
Phase										**Phase I**																**Phase II**						

Figure Description: The X-axis represents time, quarter, and the Y-axis represents the outcome during Phase I and Phase II. The dashed lines represent the median number of days between cast visits during Phase I (7.5 days, gray line) and Phase II (5.5 days, black line). The intervention aimed to decrease the median number of days between casts. The sample size, number of feet, in each quarter is displayed in the table below the figure.

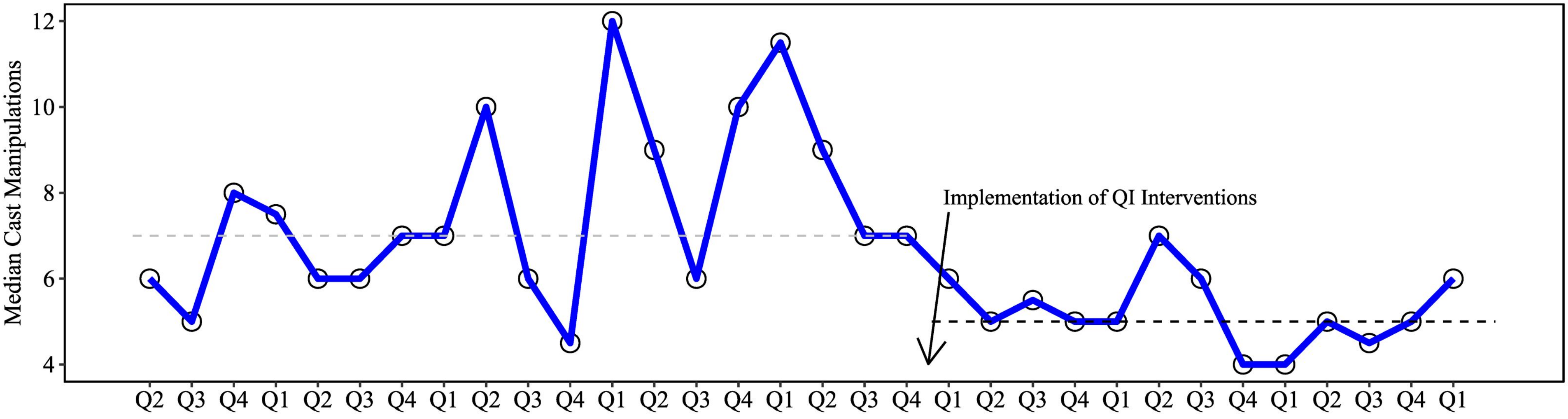

Year	**2003**			**2004**				**2005**				**2006**				**2007**				**2012**				**2013**				**2014**				**2015**
n	2	7	3	4	4	8	10	7	10	11	4	2	8	15	3	10	8	10	5	9	7	6	6	13	6	9	7	11	3	10	8	6
Phase	**Phase I**																			**Phase II**												

Figure Description: The X-axis represents time, quarter, and the Y-axis represents the outcome during Phase I and Phase II. The dashed lines represent the median number of cast manipulations per foot during Phase I (7 casts, gray line) and Phase II (5 casts, black line). A decrease in the number of cast manipulations represents a positive outcome. The sample size, number of feet, in each quarter is displayed in the table below the figure.

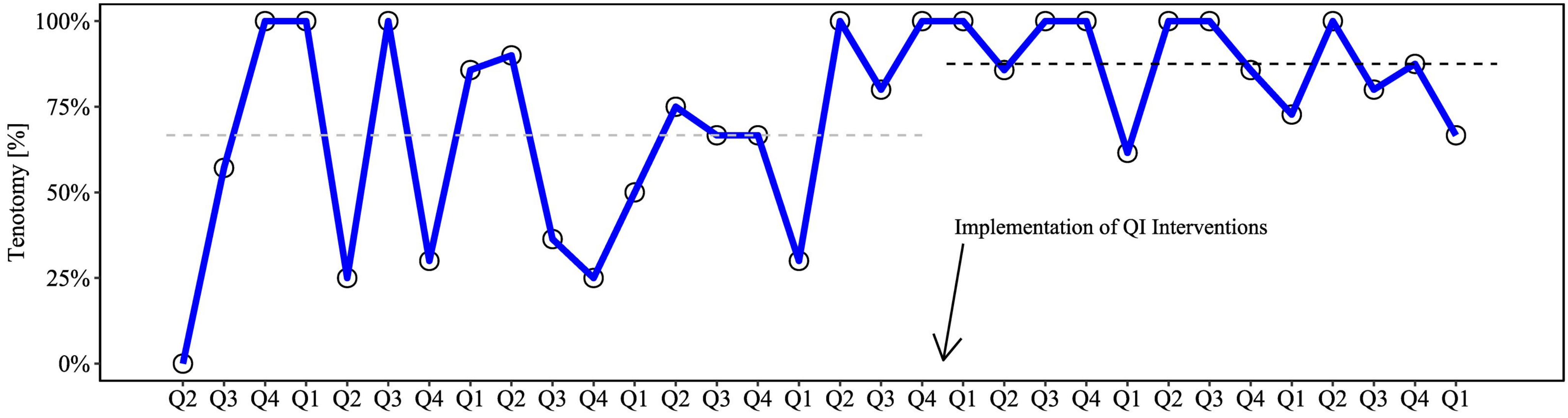

Year	**2003**	**2004**	**2005**	**2006**	**2007**	**2012**	**2013**	**2014**	**2015**
n	2 7 3	4 4 8 10	7 10 11 4	2 8 15 3	10 8 10 5	9 7 6 6	13 6 9 7	11 3 10 8	6
Phase			**Phase I**				**Phase II**		

Figure Description: The X-axis represents time, quarter, and the Y-axis represents the outcome during Phase I and Phase II. The dashed lines represent the median percentage of feet that underwent a tenotomy during Phase I (67%, gray line) and Phase II (88%, black line). We aimed to increase the percentage of feet that underwent a tenotomy in Phase II. The sample size, number of feet, in each quarter is displayed in the table below the figure.

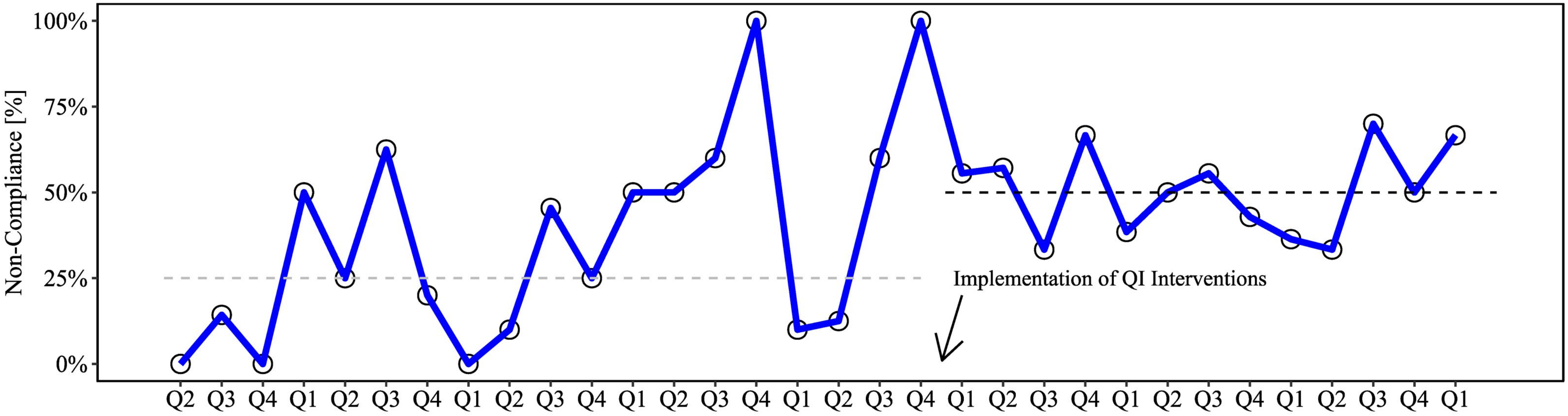

Year	**2003**			**2004**				**2005**				**2006**				**2007**				**2012**				**2013**				**2014**				**2015**
n	2	7	3	4	4	8	10	7	10	11	4	2	8	15	3	10	8	10	5	9	7	6	6	13	6	9	7	11	3	10	8	6
Phase	**Phase I**																			**Phase II**												

Figure Description: The X-axis represents time, quarter, and the Y-axis represents the outcome during Phase I and Phase II. The dashed lines represent the median percentage of feet where bracing non-compliance was noted on one or more study visits during Phase I (25%, gray line) and Phase II (50%, black line). A lower percentage of bracing non-compliance represents a positive outcome. The sample size, number of feet, in each quarter is displayed in the table below the figure.
